# Case report: Multiple approach analysis in a case of clinically assessed myotonia congenita

**DOI:** 10.3389/fgene.2024.1486977

**Published:** 2024-12-06

**Authors:** Sabrina Lucchiari, Francesco Fortunato, Giovanni Meola, Andrea Mignarri, Serena Pagliarani, Stefania Corti, Giacomo P. Comi, Dario Ronchi

**Affiliations:** ^1^ Dino Ferrari Center, Department of Pathophysiology and Transplantation, University of Milan, Milan, Italy; ^2^ Department of Neurorehabilitation Sciences, Casa di Cura Igea, Department of Biomedical Sciences for Health, Fondazione Malattie Miotoniche ETS, University of Milan, Milan, Italy; ^3^ Unit of Neurology and Neurometabolic Diseases, Department of Medical, Surgical and Neurological Sciences, University of Siena, Siena, Italy; ^4^ Foundation IRCCS Ca’ Granda Ospedale Maggiore Policlinico, Neurology Unit, Milan, Italy; ^5^ Neuromuscular and Rare Disease Unit, Department of Neuroscience, IRCCS Foundation Ca’ Granda Ospedale Maggiore Policlinico, University of Milan, Milan, Italy

**Keywords:** myotonia congenita, CLCN1, Western blot, splicing, exonic splicing silencer

## Abstract

Myotonia congenita, both in a dominant (Thomsen disease) and recessive form (Becker disease), is caused by molecular defects in *CLCN1* that encodes the major skeletal muscle chloride channel, ClC-1. This channel is important for the normal repolarization of muscle action potentials and consequent relaxation of the muscle, and its dysfunction leads to impaired muscle relaxation after voluntary or evoked contraction and muscle stiffness. More than 300 *CLCN1* pathogenic variants have been found in association with congenital myotonia, inherited as recessive or dominant traits (with complete or incomplete penetrance). In this study, we describe the case of a 44-year-old woman complaining of “leg stiffness” since the age of 20 years and presenting with transient muscle weakness, especially after sitting for several minutes, with grip myotonia and feet myotonia, cold-sensitive and warm-up. The strength was normal, but muscle hypertrophy in the lower limbs was evident. EMG myotonia was detected in all explored muscles. The patient’s father had precocious cataract correction but did not show myotonic discharges at EMG. Examination of the patient’s sons (aged 18 years and 12 years) was unremarkable. The patient started treatment with mexiletine, with improvement in grip myotonia and limb stiffness, but it was soon interrupted due to gastrointestinal disturbances. Direct sequencing of *CLCN1* identified the previously described heterozygous intronic variant c.1471 + 1G > A, which resulted in the skipping of exon 13 in the *CLCN1* muscle transcript. In addition, the rare heterozygous synonymous nucleotide change c.762C > T p.Cys254Cys was identified and predicted to alter physiological splicing. The detection of multiple splicing abnormalities leading to premature termination codons supported the *in silico* prediction. We developed a Western blot assay to assess the ClC-1 protein in muscle biopsy, and we observed that ClC-1 levels were consistently reduced in the patient’s muscle, supporting the pathogenic behavior of the variants disclosed. Overall, we report a novel case of Becker myotonia and highlight the importance of multiple levels of analysis to achieve a firm molecular diagnosis.

## Introduction

Myotonia congenita (MC) is a non-dystrophic muscular disease characterized by impaired muscle relaxation after voluntary or evoked contraction and muscle stiffness. Myotonia typically occurs after a period of rest and decreases after repetitive movement, which is the so-called warm-up phenomenon. Myotonia can be demonstrated as delayed muscle relaxation following muscle contraction or mechanical stimulation such as percussion. This channelopathy is characterized by muscle hyperexcitability, muscle stiffness, and hypertrophy, and patients often present a pseudo-athletic appearance. The clinical picture depends on whether the disease is present in the dominant (Thomsen disease) or recessive form (Becker disease) (Bryan and Alsaleem, 2024; Meyer-Kleine et al., 1995). The latter is more common and clinically more severe. The two disorders differ by the age at onset (in infancy or childhood and earlier in Becker patients), spreading of the myotonia, and a typical transient muscular weakness only present in the recessive trait (Charlet et al., 2002a; Dufner-Almeida et al., 2019).

MC, both in a dominant and recessive form, is caused by mutations in the *CLCN1* gene that encodes the major skeletal muscle chloride channel (Bryan and Alsaleem, 2024; Meyer-Kleine et al., 1995; Imbrici et al., 2015). This channel is important for the normal repolarization of muscle action potentials and consequent relaxation of the muscle. The dysfunction of this channel causes hyperexcitability of the skeletal muscle membrane and repetitive firing of muscle action potentials (Koch et al., 1992). The severity of myotonia in MC is clinically highly variable, ranging from myotonic discharges only detectable during an electromyography test (electrical myotonia) to disabling muscle stiffness at an early age (clinical myotonia) (Lin et al., 2023).

The *CLCN1* gene (GRCh38.p14; GCF_000001405.40, NG_009815.2 RefSeqGene) contains 23 exons (NM_000083.3) and is located on chromosome 7q35 (NC_000007.14) (Lorenz et al., 1994). It has been reported that there are roughly more than 300 mutations identified in the gene “http://www.hgmd.cf.ac.uk/ac/index.php.”

In this study, we present a novel case of Becker myotonia due to biallelic variants in the *CLCN1* gene, one of which was a synonymous nucleotide change of uncertain significance. The effects of the variants were evaluated in the patient’s muscle at transcript and protein levels.

## Case description

The study was approved by the Institutional Review Board of the Fondazione IRCCS Ca’ Granda Ospedale Maggiore Policlinico. The subjects involved provided written informed consent for all aspects of the study.

**FIGURE 1:**
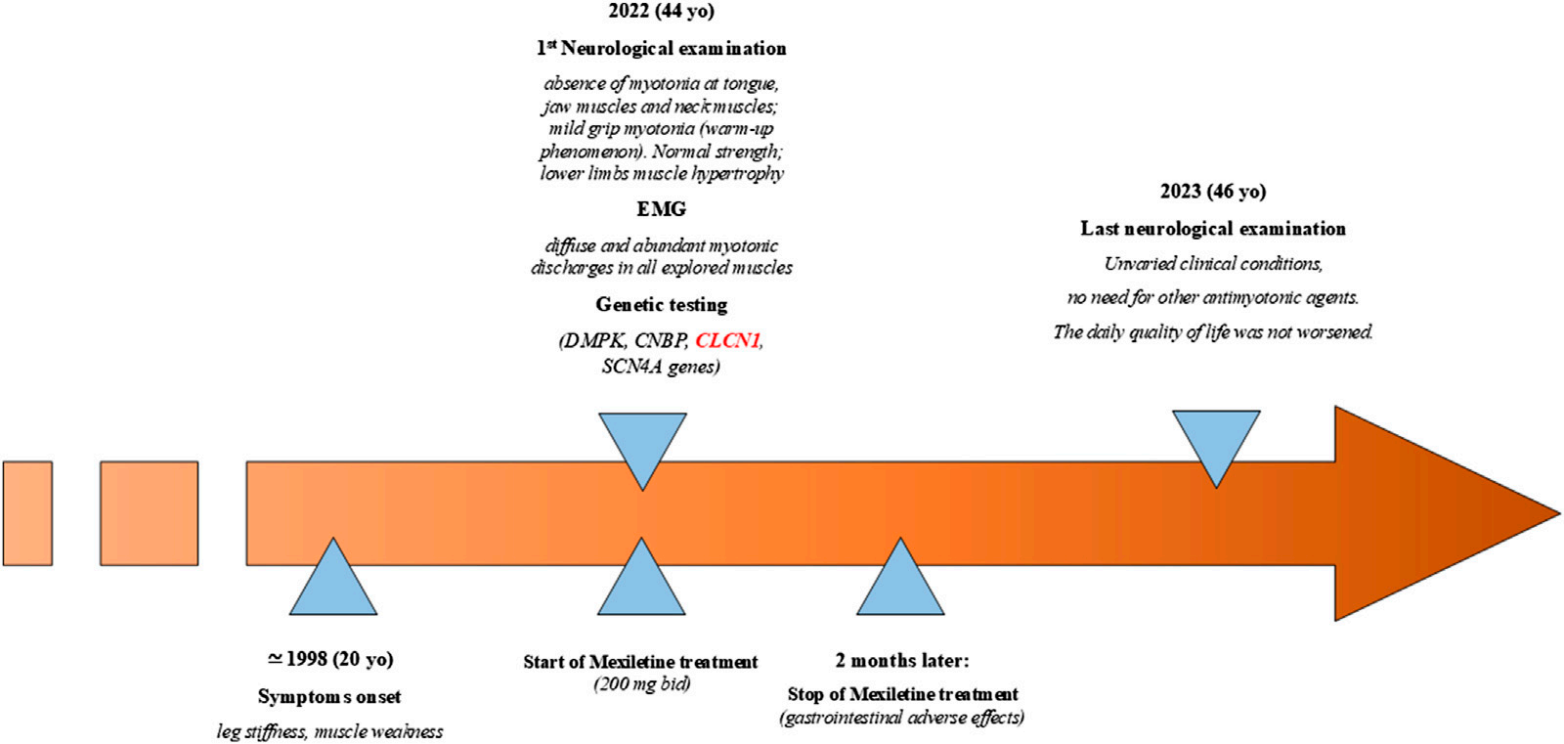
Timeline with the relevant data of the case reported.

The patient was a 44-year-old woman who came to the attention of neurologists under the suspicion for a myotonic disease; she complained since the age of 20 years (Figure 1) of “leg stiffness” and transient muscle weakness, especially after sitting for several minutes, which worsened during winter and ameliorated during summer. She also complained of grip myotonia and feet myotonia. The woman was born to non-consanguineous parents. The patient started treatment with 200 mg bid mexiletine, with improvement in grip myotonia and limb stiffness. The treatment was interrupted after 2 months due to side effects (gastrointestinal irritability).

**FIGURE 2:**
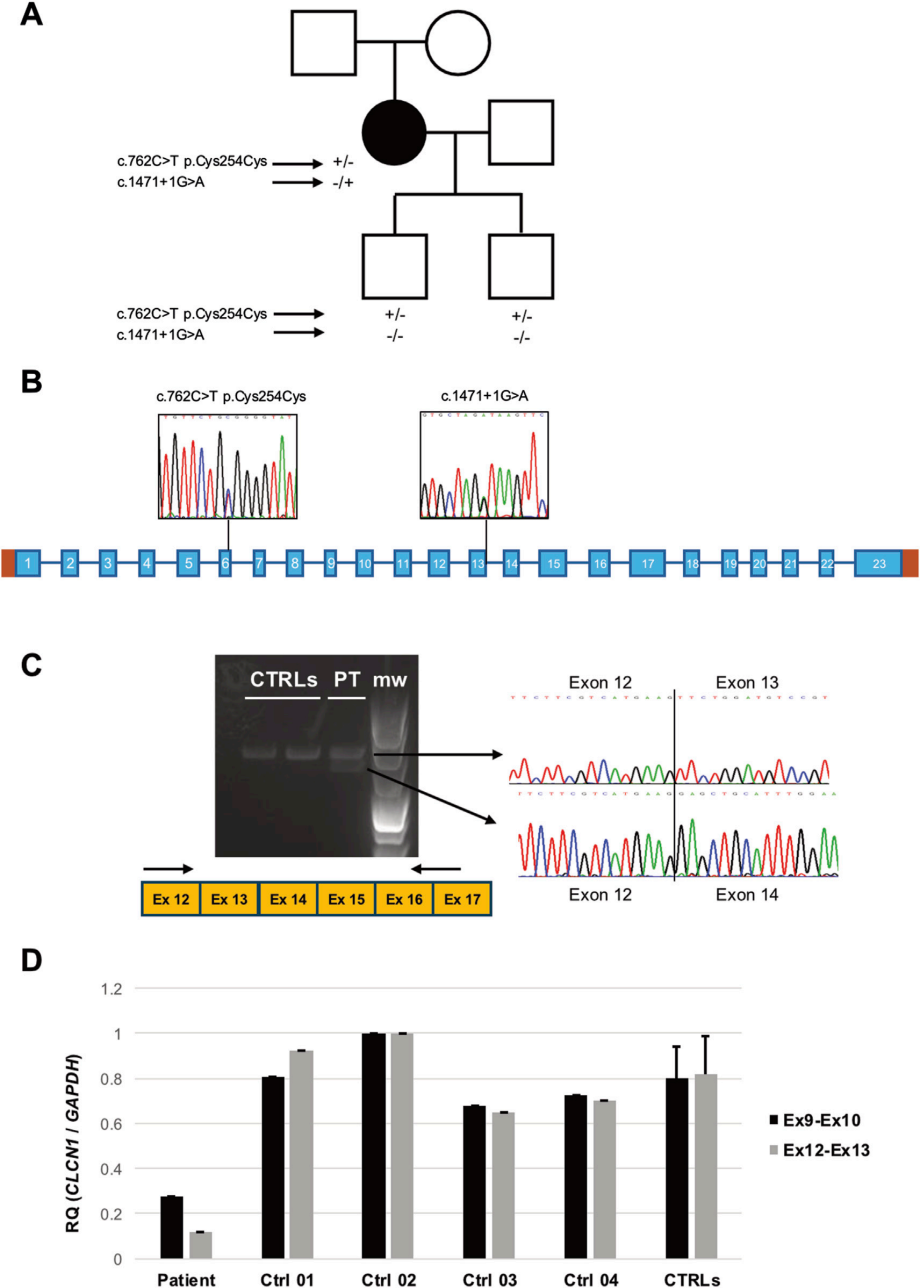
**(A)** Pedigree of the family investigated. Black symbol indicates the patient. Genotypes of the subjects who underwent molecular analysis are displayed under the respective symbols. **(B)** Scheme of the *CLCN1* gene. Sequence electropherograms displaying the variants detected in the patient are shown. **(C)** RT-PCR analysis of the *CLCN1* transcript in the patient’s (PT) and controls’ (CTRL) muscles. After subcloning, sequences of the amplicons encompassing exons 12 and 17 display the effects of the c.1471 + 1G > A variant (m.w.: molecular weight). **(D)** Quantitative RT-PCR analysis of *CLCN1* transcript levels as detected by two primer combinations targeting Ex9–10 and Ex12–13 junctions.

Neurological examination showed the absence of myotonia in the tongue, jaw muscles, and neck muscles; on the other hand, mild grip myotonia was present, which ameliorated after repeated contractions (warm-up phenomenon). The strength was normal, but muscle hypertrophy in the lower limbs was evident. The time up go (TUG) was 4–5 s, which became 1–2 s after warming up. EMG showed diffuse and abundant myotonic discharges in all explored muscles with a “dive bomber appearance,” in the absence of other alterations. Previous genetic testing ruled out DM1- and DM2-related pathological expansions, and sodium channelopathy. The patient’s father had cataract correction when he was 60 years old but did not show myotonic discharges at the EMG examination, and the mother was reported healthy. Examination of the patient’s sons (18 years old and 12 years old) was normal (Figure 2A). In 2023, the neurological examination showed unvaried clinical conditions, and the daily quality of life had not worsened; thus, there was no need to prescribe other antimyotonic agents.

Genomic DNA was extracted from peripheral blood, and the 23 coding exons and adjacent intronic sequences of the *CLCN1* gene were directly sequenced on a 3130 automated sequencer (Applied Biosystems, United States).

The *in silico* evaluation of nucleotide changes on splicing was conducted using ASSP (http://wangcomputing.com/assp/), NetGene2 (https://services.healthtech.dtu.dk/services/NetGene2-2.42/), and BDGP (https://www.fruitfly.org/seq_tools/splice.html). Eventual effects of the altered frameshift were analyzed using software available at http://bio.lundberg.gu.se/edu/translat.html.

Multiplex ligation-dependent probe amplification (MLPA) was performed to detect possible copy number variations (CNVs) on the patient’s gDNA by using Probemix P350 CLCN1-KCNJ2, purchased from MRC Holland (https://www.mrcholland.com), and following manufacturer’s instructions. PCR products were separated by capillary electrophoresis on a 3130 automated sequencer, and data were analyzed using Coffalyser.Net software.

Total RNA was extracted from a biceps specimen and reverse-transcribed, and cDNA was amplified to evidence possible aberrant splicing comparing the patient’s samples with the controls’ samples by an electrophoretic run. RT-PCR products (primers available on request), after subcloning (TOPO Invitrogen™ TOPO™ TA Cloning™ Kit) and colony picking, underwent direct sequencing. The patient’s and controls’ cDNAs were also analyzed by quantitative RT-PCR. Primers were used to target the junction between *CLCN1* exons 9 and 10 as follows: FOR_9–10 5′-GGA​ACA​AGG​ATG​CTG​TCA​CCA-3′, RC_9–10 5′-TGG​CGA​TGC​AGA​TAC​ACA​AAT​ACA-5′. For exons 12 and 13, FOR_12–13 5′-AGC​CTG​GGC​CAG​TCA​GCT-3′, RC_12–13 5′-CAG​GGT​ATG​GGC​ATA​GTG​GT-3ʹ were used. *GAPDH* was used as an internal housekeeping control. PCR amplification was performed using the Applied Biosystems 500 Real-Time PCR system (Applied Biosystems, Foster City, CA, United States) and FastStart Universal SYBR Green Master Mix (Roche Applied Science, Indianapolis, IN, United States). The relative expression levels of the targets were determined using the deltadeltaCt method in relative quantification experiments.

A 20-mg fragment from biceps biopsy was homogenized in an extraction buffer (4% SDS, 4M urea, and a protease inhibitor cocktail) using a motor-driven potter homogenizer. A measure of 50 µg of protein lysates were loaded on 9% polyacrylamide SDS gel and transferred onto a nitrocellulose membrane (Schleicher & Schuell, Keene, Nh, United States) at 70 V for 2 h. The membranes were blocked using an intercept blocking buffer, PBS (LI-COR, Lincoln, NE, United States) for 1 h at room temperature and incubated overnight at 4°C with the primary antibody for ClC-1 at 1:600 (anti-CLCN1 antibody-C-terminal, ab189857, Abcam). Actinin 1:5,000 (monoclonal antibody A7811; Sigma-Aldrich, United States) was used as an indicator of the protein loaded. The membrane was incubated with IRDye 800CW (DAR 800 V, 1:18,000) and IRDye 680RD (DAM 680R, 1:20,000) (both antibodies LI-COR, Lincoln, NE, United States). Immunoreactive bands were visualized using the Odyssey Fc system (LI-COR, Lincoln, NE, United States) and quantified by densitometry (Image Studio software).

*CLCN1* direct sequencing of the proband’s DNA identified the rare (gnomAD MAF = 0.0000002) heterozygous substitution c.1471 + 1G > A in intron 13 (Figure 2B), first described by Meyer-Kleine et al. (1995) and classified as pathogenic according to ACMG guidelines. *In silico* analysis by using ASSP, BDGP, and NetGene2 algorithms predicted the loss of the constitutive splicing donor site. To confirm this prediction, we performed *CLCN1* transcript analysis of the RNA extracted from the muscle biopsy of the patient and healthy controls. Gel electrophoresis showed the presence of an additional aberrant (shorter) band in the patient (Figure 2C). Cloning, followed by sequencing, of RT-PCR products demonstrated the existence of molecules lacking exon 13, likely resulting in the loss of a reading frame and the introduction of the premature stop codon p.Phe468Glufs*7. Quantitative RT-PCR analysis showed the significant loss of exon 13-including molecules and global reduction in *CLCN1* transcript levels in the patient’s muscle (Figure 2D). No other pathogenic or likely pathogenic variants were detected in the *CLCN1* coding sequence. MLPA analysis excluded heterozygous deletions or duplication.

Three exonic single-nucleotide variants were detected: the known benign polymorphisms c.262C>T p. (Thr87Thr) (rs6962852, exon 2, GnomAD MAF: 0.31) and c.352G>T p. (Gly118Trp) (rs10282312, exon 3, GnomAD MAF: 0.98); in homozygosis, and the heterozygous rare variant of uncertain significance c.762C>T p. Cys254Cys (rs772027125, exon 6, GnomAD MAF: 0.00003412). The variants c.762C>T and c.1471 + 1G>A were configured *in trans* as only the c.762C>T substitution was detected in the patient’s unaffected sons (Figure 2A). No other family member was available for molecular testing.

**FIGURE 3:**
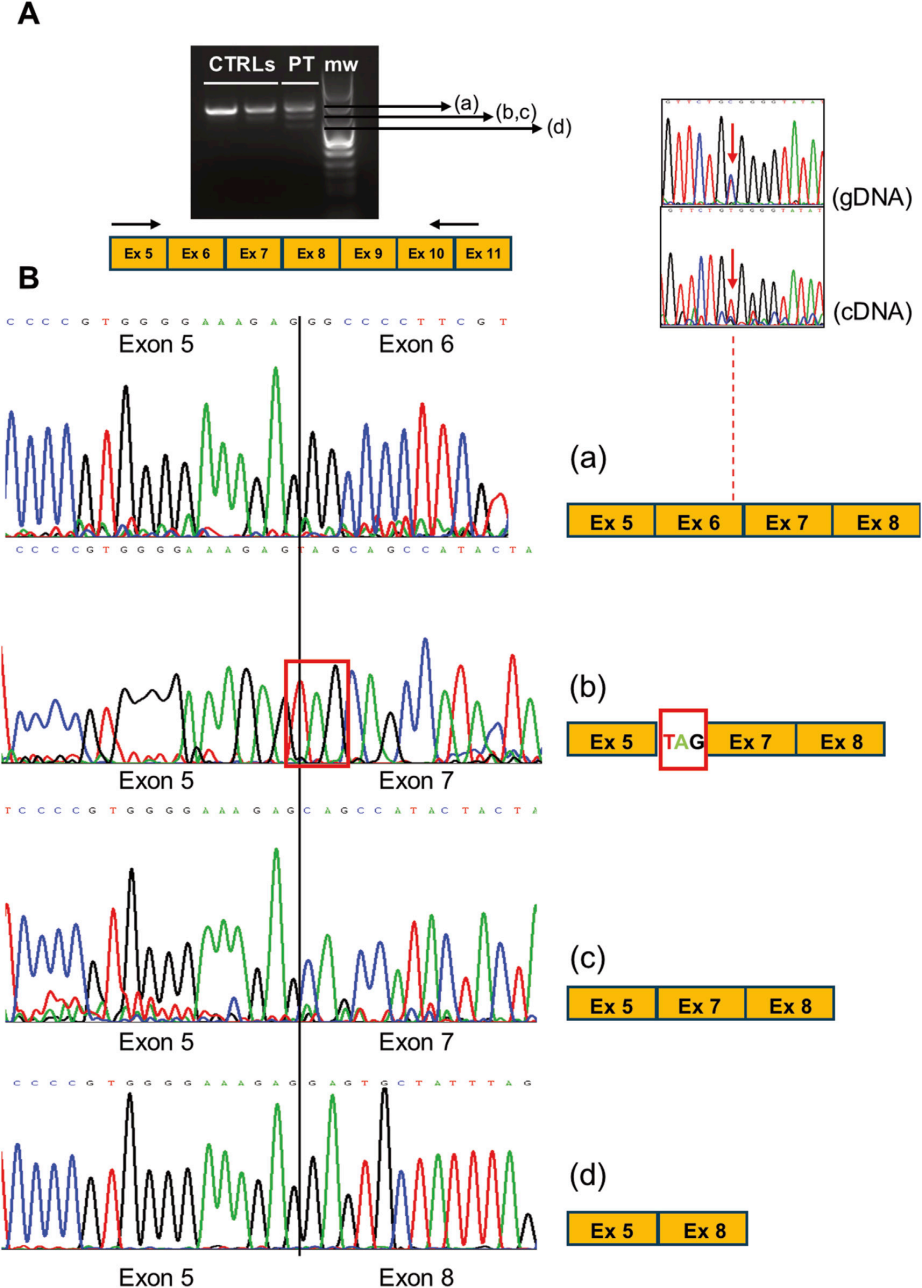
**(A)** RT-PCR analysis of *CLCN1* transcripts (ranging from exons 5 to 11 in the PT and CTRL muscles. **(B)** Electropherograms showing the sequence of the multiple splicing products obtained in the patient’s muscle, likely associated with variant c. 762C>T. The comparison of the c.762 position as detected in gDNA and cDNA shows the loss of heterozygosity (monoallelic expression) in the patient. The scheme of the exon organization is indicated at the right of each electropherogram.

We sequenced RT-PCR amplicons of the *CLCN1* transcript and noted that the c.762 position only showed the mutated “T” nucleotide, suggesting monoallelic expression in the proband’s muscle. RT-PCR analysis of transcript regions encompassing exons 5 and 11 showed four different RT-PCR products in the patient (Figure 3A). After sequencing, we observed (Figure 3B) the following: a) normally spliced molecules (including exon 6), b) molecules presenting the insertion of the trinucleotide TAG at the level of the exon 6–7 junction, c) molecules presenting the skipping of exon 6, and d) molecules displaying the skipping of exons 6 and 7. The translation of “b” and “d” misplaced products is expected to be halted prematurely, contributing to the overall reduction in the *CLCN1* transcript in the patient’s muscle, supporting the qRT-PCR findings. Full-sequence analysis of introns 5 and 6 ruled out the existence of additional rare intronic nucleotide variations.

To check the effects of the patient’s genotype at protein levels in muscle biopsies, we investigated the steady-state levels of ClC-1 by Western blot analysis by using a commercially available antibody (Figure 3). Interestingly, ClC-1 levels in the patient’s muscle were significantly reduced (about 23% of the control’s mean). The reduction in the signal (24% and 14% of the control’s mean) of samples collected from two Becker patients with known biallelic *CLCN1* defects ([c.313C>T, p.Arg105Cys]; [c.2434C>T, p.Gln812*]; [c.180 + 3A>T]; and [c.1970T>C, p. Leu657Pro]) confirmed the specificity of the assay.

**FIGURE 4:**
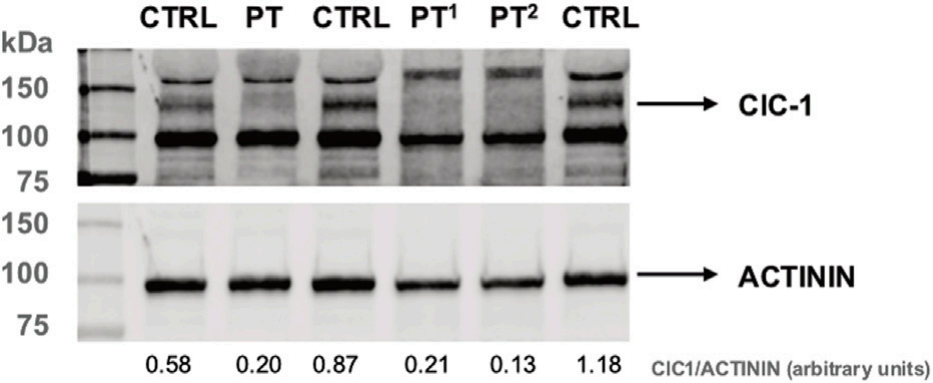
Western blot analysis of ClC-1 protein levels in PT and control muscle protein lysates. PT^1^ and PT^2^ correspond to muscle biopsies of patients harboring pathological *CLCN1* genotypes (see text). Actinin is used as a reference. The ratio between ClC-1 and α-actinin protein levels (densitometry analysis, arbitrary units) is indicated under each lane.

## Discussion

This study concerns the clinical case of a woman complaining of myotonia focalized to distal limbs, with stiffness and transient weakness since she was 20 years old, along with the classical warm-up phenomenon. Her family history was negative. Suspected as having myotonia congenita, the patient was successfully treated with 200 mg bid mexiletine until it was stopped due to severe side effects (gastrointestinal irritability). Lo Monaco et al. (2015) demonstrated that mexiletine inhibited the CMAP transient depression and that it has effects on transient weakness, a condition typically related to *CLCN1* dysfunction and reported by patients as “impairment of movement,” eventually leading to falls, which is attributed more often to muscle stiffness rather than to weakness.

The molecular analysis of the *CLCN1* gene showed that the patient carried the splice site mutation c.1471 + 1G>A. We demonstrated the effects of this variant, which shifts the translation reading frame, leading to the premature termination codon p.Phe468Glufs*7, likely followed by degradation due to nonsense-mediated decay in order to prevent the expression of the truncated protein CLC-1. This variant was first reported by Meyer-Kleine et al. (1995) and later described by others (Brugnoni et al., 2013; Ronstedt et al., 2015; Stunnenberg et al., 2018; Milla et al., 2019). Ulzi et al. (2012) described an Italian patient suffering from generalized myotonia since childhood, hypertrophy, and transient weakness, and carrying the missense p.Trp164Arg on the second allele. Tincheva et al. (2016) found c.1471 + 1G>A to be the most frequent myotonia congenita-type Becker causing mutations in the Bulgarian population. Recently, Marinakis et al. (2024) described seven patients who harbored this variant in homozygosis or in compound with other molecular defects (i.e., p.Arg894* or p.Thr550Arg) and presented comparable clinical presentations.

To our knowledge, all splice site mutations on the *CLCN1* gene, of which segregation was reported, are recessively inherited (gnomAD, LOVD https://databases.lovd.nl/shared/genes), ruling out the chance of a dominant pathological effect of the c.1471 + 1G>A allele.

After ruling out genomic rearrangements, we aimed to evaluate the potential pathogenic role of p.Cys254 as the second molecular defect in our case. It is known that nucleotide variations resulting in synonymous substitutions and located near exon–intron junctions can alter spliceosome-binding consensus sequences, perturbing physiological splicing (Dufner-Almeida et al., 2019; Lin et al., 2023; Moss et al., 2024). ESEfinder analysis of the genomic sequence harboring the c.762C>T change supported a similar scenario. In addition, *in silico* analysis using HExoSplice (http://bioinfo.univ-rouen.fr/HExoSplice/) showed that the change of TGCGGG into TGTGGG turns the sequence into an exonic splicing silencer (ESS), and this was confirmed using the SpliceAid database (http://www.introni.it/splicing.html), which located an ESS in the mutated sequence associated with the binding of a number of hnRNP factors (Supplementary Figure S1). A similar event was described by Oliveira et al. (2008) in a case of a synonymous substitution located 16 nucleotides upstream of intron 7 in the *POMGNT1* gene, able to elicit aberrant exon splicing, leading to a frameshift with the introduction of a premature stop codon. The molecular basis was the creation of the ESS motif TGTGGG, previously described by Sironi et al. as a possible inhibitor of splicing (Sironi et al., 2004).

The 3D crystallographic study of the CLC-1 channel structure (https://www.ncbi.nlm.nih.gov/Structure/pdb/6QVU) clearly shows that Cys254 is located in the loop between helixes F (amino acids 233–249) and G (amino acids 263–279) (Supplementary Figure S2A). Exon 6, which is skipped in most of the transcript molecules as a likely consequence of the c.762C>T variant, includes proline 234 that, together with proline 192, proline 486, and tyrosine 578, forms the putative Cl^−^ selectivity filter. The protein region possibly hinted by the creation of an ESS due to p.Cys254 is shown in the 3D representation (Supplementary Figure S2B), and it is a part of the complex alpha–helix system crossing the muscle cell membrane. Suetterlin et al. (2022) recently published genetic and functional data on 223 families with chloride channel myotonia and noted that, in their cohort, a disproportionately high number of variants (48%) were located in the first transmembrane domain (TM1) of the ClC-1 subunit (helixes B–I and the IJ-linker, residues 111–344). TM1 is mainly located toward the intracellular end of the intramembrane domain and hosts the selectivity filter pathway. TM1 also forms most of the subunit interfaces, suggesting that mutations in one subunit can affect the chloride conducting pathway in the neighboring subunit through this interface.

It has been previously described (Charlet et al., 2002b) that a pool of aberrantly spliced forms of *CLCN1* mRNA is present in DM1 patients, composed as follows: i) exons 5–6B–7A–7–8, ii) 5–6–6B–7A–7–8, iii) 5–8, and iv) 5–6–7–TAG. The latter has been found in both DM patients and controls without meaningful difference, and its presence is likely due to two tandem TAGs in intron 6 just upstream of exon 7, which could play the role of acceptor splice sites. As specified before, DM1 and DM2 were ruled out in our patient, so one could hypothesize that the genomic region encompassing exon 6 is particularly prone to generate different forms of aberrant splicing because it hosts sequences relevant for premature RNA processing. Unfortunately, given the rarity of this polymorphic variant, no other subject was available for further confirmation.

Fundamental support in deciphering the contributions of the two mutations found came from the availability of Western blot data. The severe reduction in ClC-1 levels supports the detrimental effect on the protein stability of the biallelic defects found in the patient. WB has never been used as a diagnostic procedure as it is time-consuming and requires the availability of muscle specimens (Wang et al., 2022), with CLC-1 expression restricted to skeletal muscle. Furthermore, affordable commercial antibodies have only become available recently (Altamura et al., 2020). Nevertheless, this method remains a gold standard for affordable protein quantification, and we encourage its use for the validation of novel *CLCN1* variants, especially those presenting uncertain or ambiguous pathogenic significance.

This work is indicative of the benefits of the simultaneous use of multiple techniques for studying disease-causing mutations. A multilevel approach is obviously desirable in most cases, and it is crucial for establishing the effects of (novel) nucleotide variants on protein products. It offered us the opportunity to expand the molecular and biochemical findings in our patient. Furthermore, it shed light on the potential pathological role of synonymous variants, which are worthy of deep investigations.

## Data Availability

The datasets presented in this article are not readily available because of ethical and privacy restrictions. Requests to access the datasets should be directed to the corresponding author.
