# Supplementary material for: Case report: Multiple approach analysis in a case of clinically assessed myotonia congenita
Source: Front Genet. 2024 Dec 6;15:1486977. doi: 10.3389/fgene.2024.1486977 (PMC11659273; doi:10.3389/fgene.2024.1486977)
Supplement: Supplementary file 1 [file DataSheet1.pdf]

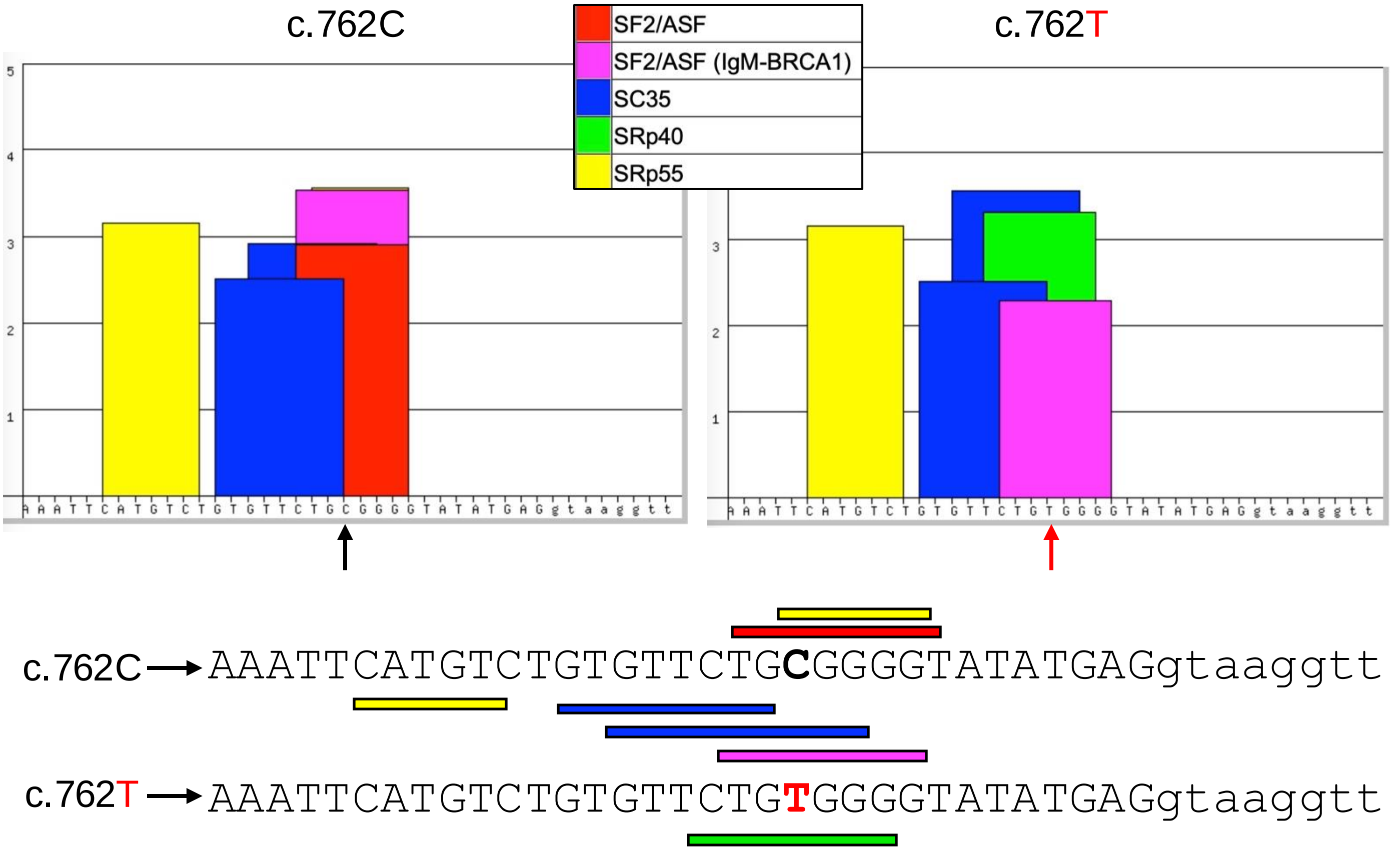

Supplementary Figure 1

ESE finder analysis of the genomic sequence surrounding the c.752 position in CLCN1 wildtype (c.762C) or mutated (c.762T) alleles. The predicted binding regions of the splicing factors which are specific for the wildtype and mutated alleles are indicated above and under the sequences, respectively.

A

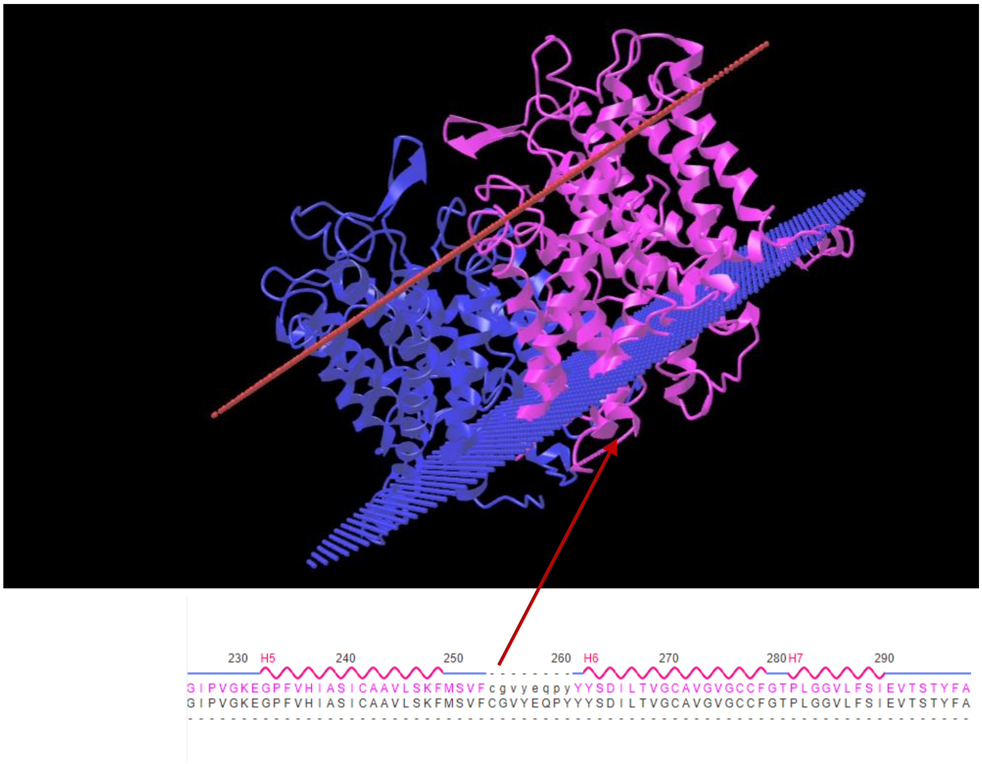

B

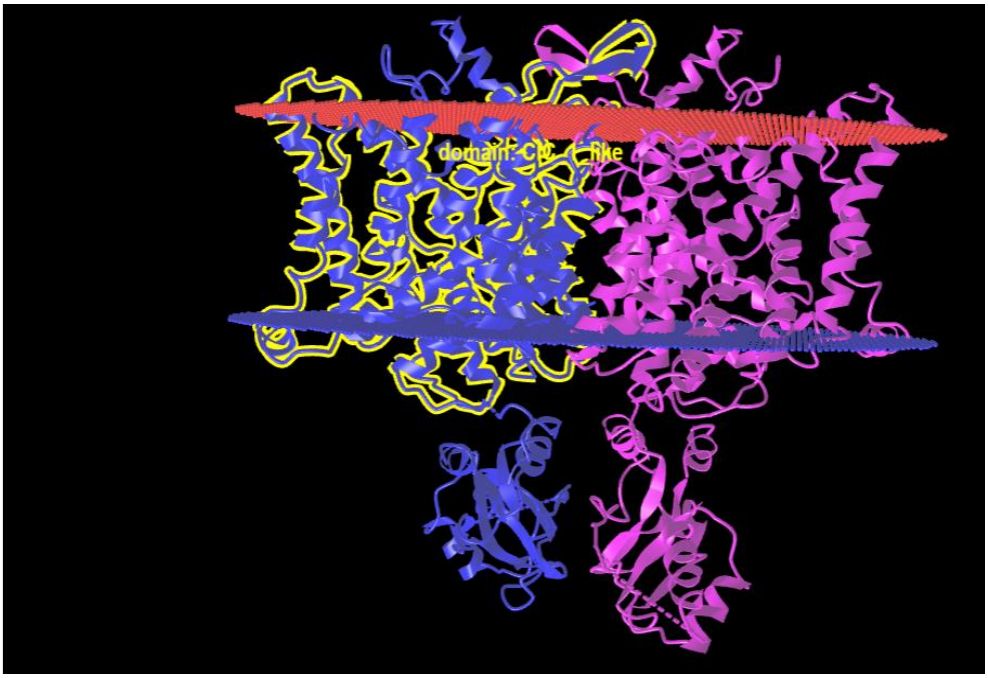

Supplementary Figure 2

CryoEM structure of the human chloride channel showing: (**A**) Cysteine 254 located at position four of the inter-helix loop, as indicated by the primary structure below the image (PDB ID:6QV6); (**B**) the domains likely deleted as a consequence of the c.762C>T change are highlighted in yellow (PDB ID:6QVU).

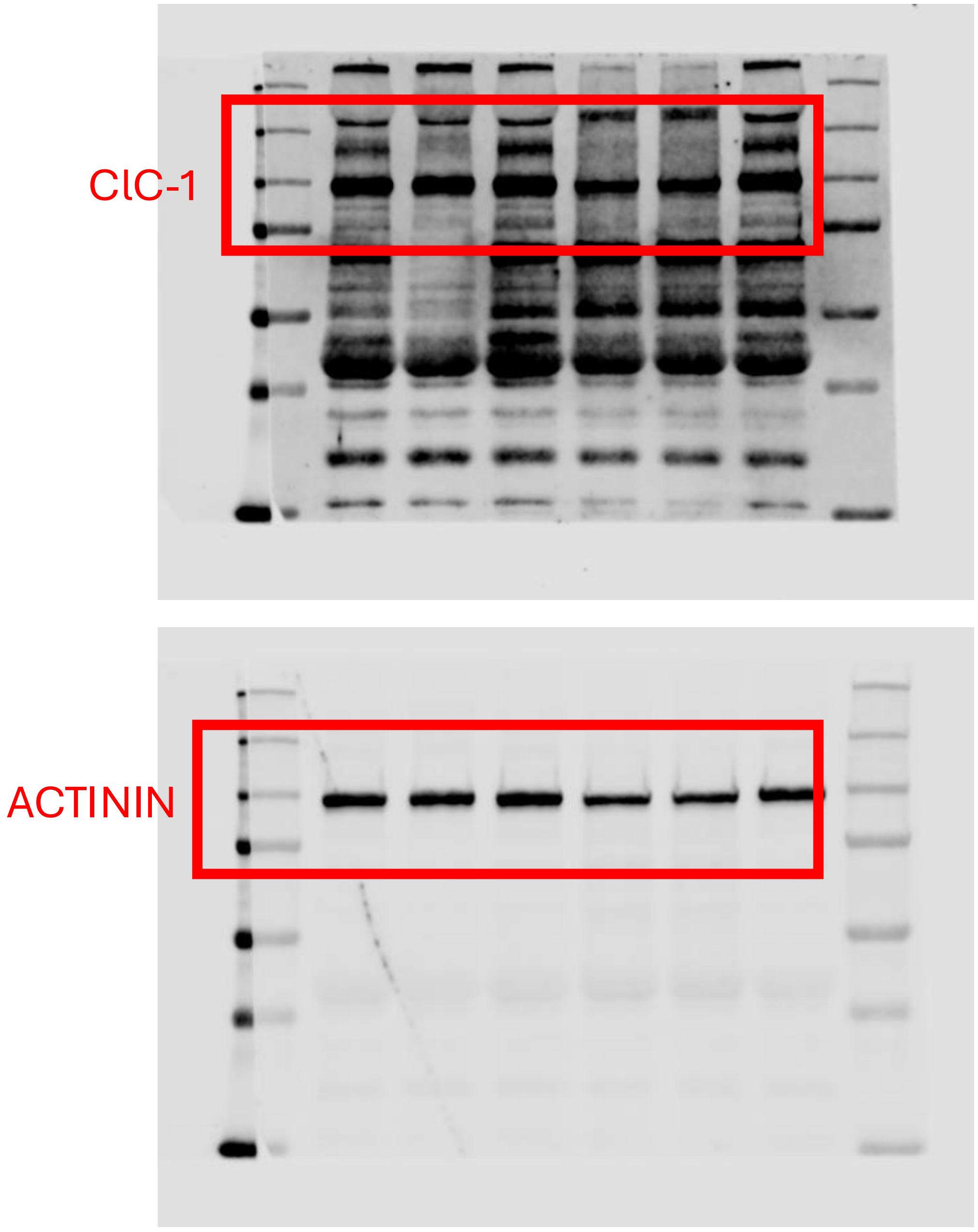

Supplementary Figure 3
Full scan of the entire original gels presented as cropped images in Figure 3.
